# Peptide-Based Agents for Cancer Treatment: Current Applications and Future Directions

**DOI:** 10.3390/ijms241612931

**Published:** 2023-08-18

**Authors:** Nguyễn Thị Thanh Nhàn, Tohru Yamada, Kaori H. Yamada

**Affiliations:** 1 Department of Pharmacology & Regenerative Medicine, University of Illinois College of Medicine, Chicago, IL 60612, USA; nhann@uic.edu; 2 Department of Surgery, Division of Surgical Oncology, University of Illinois College of Medicine, Chicago, IL 60612, USA; tohru@uic.edu; 3 Richard & Loan Hill Department of Biomedical Engineering, University of Illinois College of Engineering, Chicago, IL 60607, USA; 4 Department of Ophthalmology & Visual Sciences, University of Illinois College of Medicine, Chicago, IL 60612, USA

**Keywords:** amino acid, tumor, targeting delivery, diagnosis, imaging, chemical modification

## Abstract

Peptide-based strategies have received an enormous amount of attention because of their specificity and applicability. Their specificity and tumor-targeting ability are applied to diagnosis and treatment for cancer patients. In this review, we will summarize recent advancements and future perspectives on peptide-based strategies for cancer treatment. The literature search was conducted to identify relevant articles for peptide-based strategies for cancer treatment. It was performed using PubMed for articles in English until June 2023. Information on clinical trials was also obtained from ClinicalTrial.gov. Given that peptide-based strategies have several advantages such as targeted delivery to the diseased area, personalized designs, relatively small sizes, and simple production process, bioactive peptides having anti-cancer activities (anti-cancer peptides or ACPs) have been tested in pre-clinical settings and clinical trials. The capability of peptides for tumor targeting is essentially useful for peptide–drug conjugates (PDCs), diagnosis, and image-guided surgery. Immunomodulation with peptide vaccines has been extensively tested in clinical trials. Despite such advantages, FDA-approved peptide agents for solid cancer are still limited. This review will provide a detailed overview of current approaches, design strategies, routes of administration, and new technological advancements. We will highlight the success and limitations of peptide-based therapies for cancer treatment.

## 1. Introduction

Cancer represents a profound worldwide public health challenge, demanding significant attention and resources [1]. According to a recent study conducted in 21 countries across five continents, cancer emerges as the primary reason for mortality in numerous nations [2]. Major challenges in cancer treatment include the emergence of multidrug resistance and the scarcity of tumor-specific therapies that exhibit minimal side effects. Cancer therapy has undergone significant advancements, but the need for more effective and targeted treatments remains [3]. One of the key aspects of cancer therapy is the targeted delivery of chemotherapeutic agents to cancer cells, maximizing treatment effectiveness while minimizing harm to healthy tissue. Bioactive peptides have gained attention due to their potential anticancer properties. Peptide-based approaches offer several advantages in cancer treatment, including enhanced specificity, reduced toxicity to normal tissues, and versatility in targeting various molecular pathways involved in cancer progression [4,5,6,7].

Diverse natural and modified peptides have been extensively studied and acquired, spanning numerous therapeutic areas. Therapeutic peptides act as hormones, growth factors, neurotransmitters, ion channel ligands, or anti-infective agents. They possess high affinity and specificity when binding to cell surface receptors, triggering specific intracellular effects. In terms of their mode of action, therapeutic peptides share similarities with biologics like therapeutic proteins and antibodies, offering targeted and specific therapeutic approaches. Peptides have certain drawbacks compared to antibodies, including a shorter half-life due to rapid excretion and susceptibility to protease degradation. However, peptides offer advantages such as low cost, the ability to penetrate deep tissue, efficient internalization into cells, lower immunogenicity and reduced toxicity towards bone marrow and the liver, and ease of modification using chemical methods, setting them apart from antibodies [8,9].

In this review, we will describe the peptide-based approaches for cancer diagnosis and treatment, the design strategies, and summarize peptide-based-cancer diagnosis and therapies in clinical and pre-clinical to provide an overview of peptide-based agents for cancer treatment.

## 2. Peptide-Based Approaches for Cancer Treatment

Peptide-based therapeutic approaches for cancer treatment encompass a wide range of strategies that leverage the unique properties of peptides to target and combat cancer cells.

### 2.1. Anti-Cancer Peptides (ACPs)

ACPs have undergone recent enhancements to be transformed into drugs and vaccines, subsequently undergoing evaluation through different stages of clinical trials [10]. ACPs are short bioactive peptides composed of 10–60 amino acids, which exert their therapeutic effects through various mechanisms that specifically target cancer cells while sparing normal cells. Common mechanisms of action for anti-cancer peptides are apoptosis induction, membrane disruption, angiogenesis inhibition, signaling pathway modulation, and immunomodulation. In addition, ACPs can interfere with key signaling pathways that promote cancer cell survival, proliferation, and metastasis. These peptides may target proteins involved in cell cycle regulation, growth factor signaling, or survival pathways, leading to the inhibition of cancer cell growth and survival.

#### 2.1.1. Induction of Apoptosis

**Figure 1:**
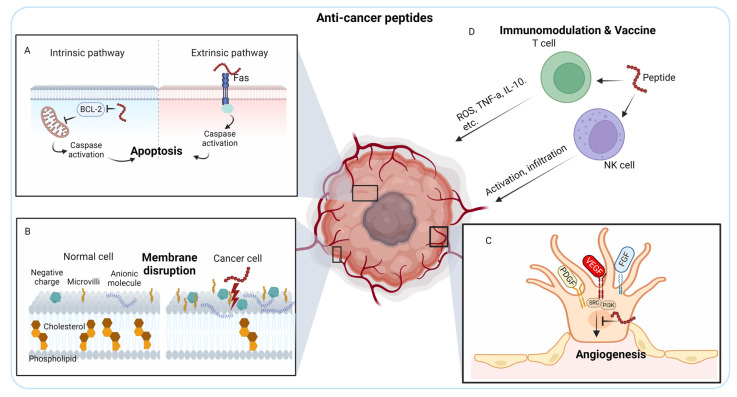
Mechanisms of anti-cancer peptides, highlighted for each mechanism indicated by boxes and their magnified boxes with schemes. (**A**) Apoptosis induction. (**B**) Membrane disruption. (**C**) Inhibition of tumor angiogenesis. (**D**) Immunomodulation and peptide vaccine.

ACPs can trigger programmed cell death, known as apoptosis, in cancer cells. They can directly target specific intracellular components involved in apoptosis regulation, such as mitochondrial membranes or caspases, leading to the activation of apoptotic pathways. This results in the controlled elimination of cancer cells. Out of the various bioactivities, the apoptosis pathway is recognized as the most efficacious strategy in non-surgical cancer therapies because of its ability to cause minimal inflammation and damage to the targeted regions [11,12]. The anti-apoptotic B-cell lymphoma 2 (BCL-2) family proteins primarily regulate the intrinsic apoptosis pathway, whereas the extrinsic apoptosis pathway involves Tumor necrosis factor (TNF) receptor (TNFR), FAS (CD95), and Death receptor 3 (DR3)/WSL [13] (Figure 1A). Peptide-based cancer therapy can target both apoptosis pathways. Antimicrobial peptides from *Anabas testudineus* AtMP1 and AtMP2 (Table 1 for peptides tested in pre-clinical settings) induce apoptosis of breast cancer cells MCF7 and MDA-MB-231 by down-regulating *BCL-2* gene [14]. In contrast, another antimicrobial peptide from Nile tilapia (*Oreochromis niloticus*), MSP-4, induces the apoptosis of osteosarcoma MG63 cells through a Fas/FasL-mediated pathway [15].

A peptide fragment, p28 (NSC745104) (CDG Therapeutics Inc., Elk Grove Village, IL, USA) (Table 2 for peptides tested in clinical trials) derived from bacterial protein azurin also induces cell cycle arrest in various types of human cancer cells [16,17]. Cupredoxin azurin is secreted by the opportunistic pathogen *Pseudomonas aeruginosa* in response to increasing numbers of and proximity to cancer cells [18,19,20,21,22]. Secreted azurin preferentially enters a variety of solid tumor cell lines including breast cancers and induces p53-mediated apoptosis [18,19,23,24,25]. A fragment of azurin, amino acids 50 to 77 (p28), is responsible for azurin’s preferential penetration and anti-proliferative activity [16,26]. As a single therapeutic agent, p28 (NSC745104) was tested in two Phase I clinical trials (NCT00914914, NCT01975116) and granted the FDA Orphan Drug and Rare Pediatric Disease Designations since it showed preliminary efficacy without apparent adverse effects, toxicity, or immunogenicity in patients with advanced solid tumors and in pediatric patients with recurrent and refractory central nervus system (CNS) tumors (NCI and Pediatric Brain Tumor Consortium) [27,28,29].

**Table 1:** Examples of peptides in pre-clinical tests.

Name	Sequence	Effects	Refs.
AtMP1	THPPTTTTTTTTTTTTTAAPATTT	Apoptosis	[14]
AtMP2	TGIATSGLATFTLHTGSLAPAT	Apoptosis	[14]
MSP-4	FIHHIIGGLFSAGKAIHRLIRRRRR	Apoptosis	[15]
peptide 8.6	Ac-MCTIDFDEYRFRKT-NH_2_	Apoptosis	[30]
HPRP-A1-TAT	Ac-FKKLKKLFSKLWN WK-RKKRRQRRR-NH_2_	Membrane disruption	[31]
melittin	NH_2_-GIGAVLKVLTTGLPALISWIKRKRQQ-NH_2_	Membrane disruption	[32]
QR-KLU	QKRKRKKSRY-KLUKLUKKLUKLUK	Angiogenesis inhibition	[33]
KV11	YTMNPRKLFDY	Angiogenesis inhibition	[34]
KAI	SRGTPVDERLFLIVRVTVQLSHP-NH_2_	Angiogenesis inhibition	[35,36,37]
LFcinB_26–36_	RRWQWRMKKLG	Immunomodulation	[38,39]
LFcinB_17–41_	FKCRRWQWRMKKLGAPSITCVRRAF	Immunomodulation	[38,39]
CREKA	CREKA	Tumor-homing	[40]
RGD-4C	CDCRGDCFCG	Tumor-homing	[41]
c(RGDyK)	c(RGDyK)	Tumor-homing	[42]
CCK8	DY(SO_3_H)MGWMDF-NH_2_	Tumor-homing	[43]
TAT	YGRKKRRQRRR	CPP	[44]
Penetratin	RQIKIWFQNRRMKWKK	CPP	[45,46]
Transportan	GWTLNSAGYLLGKINLKALAALAKKIL-NH_2_	CPP	[47]
M13	AGYLLGKINLKACAALAKKCL	CPP	[48,49]
pA	cNGQGEQc	Targeting integrins	[50]
NKTP-3	kkRRuPLBIUBDPVdRRKrgerppr	Inhibits tumor growth	[51]
KRpep-2d	Ac-RRRRCPLYISYDPVCRRRR-NH2	Inhibits tumor growth	[52]
HVGGSSV	HVGGSSV	Imaging	[53,54]
X4-2-6	LLFVITLPFWAVDAVANWYFGNDD-PEG27	Preventing metastasis	[55]
CLT1	CGLIIQKNEC	Tumor-homing	[56]
C5C2	SSVVQSTGHMQSKVYESVLALSAEVQAAR-NH_2_	BBB permeabilization	[57]
HAV6	Ac-SHAVSS-NH_2_	BBB permeabilization	[58,59,60]
K16ApoE	KKKKKKKKKKKKKKKKLRVRLASHLRKLRKRLLRDA	RMT	[61]
AEP	LRKLRKRLLR	RMT	[62]
RAP12	EAKIEKHNHYQK	RMT	[63]
melanotransferrin (MTf)-derived peptide	DSSHAFTLDELR	RMT	[64]
Peptide-22 (VH434)	Ac-[CMPRLRGC]c-NH2	RMT	[49,65]
L57	TWPKHFDKHTFYSILKLGKH-OH	RMT	[66]
M1	TFYGGRPKRNNFLRGIR	RMT	[67]
LRPep2	HPWCCGLRLDLR	RMT	[68]
TfR-T12	THRPPMWSPVWP	RMT	[69]
T7	HAIYPRH	RMT	[69]
B6	G GHKAKGPRKLGS	RMT	[70]
CRT peptide	CRTIGPSVC	RMT	[71]
NanoLigand Carriers (NLC)	CGYRPVHNIRGHWAPG	RMT	[72]
Leptin30	YQQILTSMPSRNVIQISNDLENLRDLLHVL	RMT	[73,74]
g21	TLIKTIVTRINDISHTQSVSA	RMT	[75]
A7R	ATWLPPR	RMT	[76,77]
IL-13p	TAMRAVDKLLLHLKKLFREGQFNRNFESIIICRDRT	RMT	[78]
Pep-1	CGEMGWVRC	RMT	[79,80]
G7	GFtGFLS	RMT	[81,82]
RVG-9R	YTIWMPENPRPGTPCDIFTNSRGKRASNGGGGRRRRRRRR	RMT	[83]
RDP	MGKSVRTWNEIIPSKGCLRVGGRCHPHVNGGG-RRRRRRRRR	RMT	[84]
39 mer RDP	KSVRTWNEIIPSKGCLR VGGRCHPH VNGGGRRRRRRRRR	RMT	[85]
KC2S	YTKTWCDGFCSSRGKRIDLG	RMT	[86]
CDX	FKESWREARGTRIERG	RMT	[87,88]
MiniAp-4	H-[Dap]KAPETALD-NH_2_	RMT	[89]
TGN	TGNYKALHPHNG	RMT	[90]
SynB1	RGGRLSYSRRRFSTSTGR	AMT	[91]
CAQK	CAQK	AMT	[92]
G23	HLNILSTLWKYR	AMT	[93,94]
PepH3	AGILKRW	AMT	[95]
*N*-methyl phenylalanine-rich peptide *	*N*-MePhe-(*N*-MePhe)_3_-CONH_2_	AMT	[96]
phenylproline tetrapeptide *	(PhPro)_4_	AMT	[97]
NegPep	SGTQEEY	AMT	[98]
Porphyrin	AGILKRWK-NH_2_	AMT	[99]
NFL-TBS.40–63	YSSYSAPVSSSLSVRRSYSSSSGS	AMT	[100,101]
LMWP	CVSRRRRRRGGRRRR	AMT	[102]

Small letter indicates D amino acids. C indicates cyclized peptide. * indicates peptides described as 3 letter code and functional groups. Ph, Me, CONH_2_, NH_2_, SO_3_H, OH, Ac are functional groups. Some modifications may not be depicted here such as disulfide bond or PEGylation.

ATSP-7041 (Aileron Therapeutics, Watertown, MA, USA) is also activating the p53 pathway by interacting and inhibiting mouse double minute 2 (MDM2) and MDMX (MDM4) [131]. MDM2 is an E3 ubiquitin ligase that inhibits p53 by targeting it for degradation. MDMX inhibits p53’s transactivation activity and promotes MDM2 activity via direct protein–protein interactions. ATSP-7041 and its derivative ALRN-6924 were developed from N-terminal α-helical domain of the p53 tumor suppressor protein, which directly binds to MDM2/MDMX [132] and inhibits growth of solid tumors and lymphomas [133,134,135]. ALRN-6924 was tested in Phase I/II clinical trial for patients with solid tumors and lymphomas (NCT02264613). ALRN-6924 was well-tolerated as there was evidence of single-agent anti-tumor activity, including complete and partial responses [103,104]. However, Phase Ib chemoprotection trial in patients with p53-mutated breast cancer (NCT05622058) was terminated as patients experienced severe, grade 4 neutropenia and alopecia, failing to meet the main end points of the trial [136].

Additionally, some ACPs can interact with DNA molecules within cancer cells. They can bind to the DNA helix, induce conformational changes, or interfere with DNA replication, transcription, or repair processes. This disruption of DNA integrity can lead to DNA damage, genomic instability, and subsequent cell cycle arrest or cell death. For instance, White et al. discovered a peptide that specifically targets the C-terminal domain of breast cancer-associated protein 1 (BRCA1) in breast cancer, which altered the DNA damage response [30].

#### 2.1.2. Membrane Disruption

Negatively charged, low cholesterol, and aberrant microvilli content cancer cell surfaces could facilitate the specific activity of ACPs against cancer cells [137,138]. (i) In healthy cells, negatively charged phospholipids are mainly found in the inner membrane leaflets, but in cancer cells, this asymmetry is disrupted, causing overexpression of negatively charged phosphatidylserine on the cell membrane surface (Figure 1B). Additionally, other anionic molecules, such as O-glycosylated mucins, and the glycosaminoglycan side chains mainly in the form of heparin sulfate, further increasing the negative charges on cancer cells. (ii) Furthermore, in healthy cell membranes, cholesterol serves as a crucial regulator of fluidity, contributing to the inhibition of cationic peptide entry or translocation. The lower cholesterol content in cancer cell membranes compromises this protective mechanism, thereby augmenting their vulnerability to the cytolytic effects of ACPs (Figure 1B). Most membrane-disrupting ACPs possess amphipathic properties, meaning they have both hydrophobic and hydrophilic regions. These peptides can interact with the lipid bilayer of cancer cell membranes, causing disruption and destabilization. This disruption can lead to increased permeability, leakage of intracellular components, and, ultimately, cell lysis. (iii) Moreover, the increased abundance and aberrant morphology of microvilli on cancer cells augment the cell surface area and contact with ACPs, further enhancing their interaction with these peptides.

One example of ACPs targeting cancer cell membranes is HPRP-A1-TAT [31], a hybrid peptide that can destroy the cell membrane to cause rapid leakage of cytoplasmic contents and has a strong anti-cancer activity. The IC_50_ value of this ACP in melanoma, gastric, liver, and cervical cancer cells is less than 10 µM [31]. Another example of membrane-disrupting peptide is melittin [32]. To reduce the hemolytic side effect of melittin, encapsulating melittin in hydrogel by conjugating with hydrogel self-assembling peptide RADA32 and loading doxorubicin reduced side effects and selectively inhibited tumor growth, recruited activated natural killer (NK) cells in the primary melanoma tumor, activated dendritic cells, and generated cytotoxic T-cells against remaining tumors [139].

#### 2.1.3. Inhibition of Tumor Angiogenesis

Angiogenesis is the process where new capillaries are formed from pre-existing blood vessels, and it has a vital function in cancer by providing oxygen and nutrients necessary for tumor growth and metastasis [140,141]. Angiogenesis is controlled by various signaling pathways, such as vascular endothelial growth factor (VEGF), fibroblast growth factor (FGF), platelet-derived growth factor (PDGF), and angiopoietins [141]. Of these pathways, VEGF holds particular significance. In both preclinical tumor models and human cancer patients, the exclusive inhibition of VEGF by antibodies or small molecules has demonstrated notable effectiveness in antiangiogenic therapy, yielding positive outcomes [142]. However, resistance to VEGF inhibitors occurs through various mechanisms [143]. ACPs are studied as alternative approaches which suppress angiogenesis by targeting and inhibiting specific signaling pathways involved in angiogenesis, thereby reducing the blood supply to tumors and impeding their progression (Figure 1C). Wang et al. developed a VEGFR targeting PDC, which suppresses tumor angiogenesis in transcatheter arterial chemoembolization (TACE) model for hepatocellular carcinoma therapy [33]. KV11, an 11-amino acid peptide derived from apolipoprotein A (ApoA), inhibits angiogenesis both in vitro and in vivo, specifically targeting the c-Src/ERK signaling pathways [34].

Another example of the small peptide angiogenesis inhibitor, KAI, was designed to inhibit KIF13B-mediated VEGFR2 trafficking to the cell surface, thereby inhibiting receiving VEGF [144,145]. The peptide KAI, a kinesin-derived angiogenesis inhibitor, inhibits trafficking of VEGFR2 from the Golgi apparatus and recycling of internalized VEGFR2 and inhibits pathological angiogenesis and vascular leakage in wet age-related macular degeneration, blinding eye disease [35,36]. KAI also successfully penetrates the cell membrane by utilizing cationic residues, resulting in inhibiting tumor angiogenesis and tumor growth [144] and metastasis in cancer models in mice [37].

#### 2.1.4. Immunomodulation

Cancer cells can evade the immune system’s detection and response mechanisms, allowing them to escape elimination [146,147]. Immunotherapies employ various approaches to target immune cells and enhance their ability to kill cancer [148]. ACPs can modulate the immune response against cancer cells by stimulating the activation and proliferation of immune cells, such as T cells and NK cells, leading to enhanced recognition and elimination of cancer cells by the immune system (Figure 1D). Additionally, ACPs may modulate immune checkpoints, enhancing the antitumor immune response [149,150]. Bovine lactoferrin (LfcinB), a peptide derived from lactoferrin, hinders the growth of head and neck squamous cell carcinoma by inducing increased lymphocyte infiltration to inhibit head and neck squamous cell carcinoma in vivo [38,39].

#### 2.1.5. Peptide Vaccine

Among immunotherapies, in addition to immunomodulators, peptide vaccines play a crucial role in educating the immune system to generate anti-cancer activity. By stimulating specific immune responses against cancer cells, peptide vaccines enhance the body’s capacity to recognize and effectively target them. The efficacy of cancer vaccines is closely linked to the recognition of tumor antigens by T lymphocytes. The ideal antigen for cancer vaccines should exhibit exclusive expression in cancer cells and possess high immunogenicity [151]. Peptide-based cancer vaccines typically consist of 20–30 amino acids containing specific epitopes from highly immunogenic antigens, aiming to induce the desired immune response. Compared to other vaccine types, peptide vaccines offer several benefits, particularly regarding safety and production simplicity [152]. The E75 peptide breast cancer vaccine (Her2 p369–p377) [153] with polyactin A has been demonstrated to increase CD4^+^ and CD8^+^ T lymphocytes, enhance splenocyte proliferation, and elevate interferon-γ levels [154]. Nelipepimut-S (E75, HER2/Neu, NeuVax) (SELLAS Life Sciences, New York, NY, USA) has been tested in clinical trials (NCT00841399 [106], NCT00091286 [107], NCT00791037 [107], NCT01532960 [108]). However, the Phase III clinical trial (NCT01479244) failed to show the difference in disease-free survival between Nelipepimut-S and placebo [109]. Combination therapies with trastuzumab Phase IIb (NCT01570036) showed clinical benefit in patients with triple-negative breast cancer (TNBC) [110].

### 2.2. Tumor-Homing Peptides

Tumor-homing peptides (THPs) are oligopeptides, usually consisting of 30 or fewer amino acids that are efficiently and specifically incorporated into tumor cells [155]. They are designed to be tumor cell-specific to enhance the internalization of small-molecule drugs or chemotherapeutic agents by creating PDC, enabling targeted delivery of therapeutic payloads to cancer cells [156]. Additionally, nanoparticles or liposomes can be modified with peptides to deliver the chemotherapeutic agents loaded in the nanoparticles/liposomes into tumor cells. [156]. CREKA (CREKA) can recognize clotted plasma proteins and selectively homes to tumors, where it binds to vessel walls and tumor stroma [40]. This peptide successfully amplified nanoparticles home to tumors in vivo [40].

RGD peptide (Arg-Gly-Asp), derived from integrin-binding motif from extracellular matrix proteins [157], specifically targets integrin αVβ3 receptors, which is highly expressed in several types of tumors. RGD is widely tested for potential use in diagnostic imaging [158,159] and therapies by conjugating with drugs or coating nanoparticles/liposomes [160]. The αv integrin-specific internalizing RGD (iRGD) (CRGD R/K GP D/E C) [161] (was fused with exosome membrane protein Lamp2 to make tumor-targeting exosome [162]. This strategy enabled effective and targeted delivery of drugs (e.g., doxorubicin) to breast cancer cells expressing αv integrin, resulting in the inhibition of tumor growth in mice [162]. The iRGD (Cend-1, Cend Therapeutics, San Diego, CA, USA) has been tested in clinical trials for the treatment of metastatic pancreatic cancer [111]. RGD peptides, including RGD-4C (ACDCRGDCFCG) peptide [41], c(RGDyK) [42], and small molecules Cilengitide™ (cRGDfV, EMD 121974) (ICENI Pharma, Edinburgh, UK) [163], target tumor vasculature by binding overexpressed αvβ3 integrin in the angiogenic endothelial cells and inhibit angiogenesis [164,165,166]. Cilengitide has been tested in clinical trials. However, a multicenter randomized open-label Phase III clinical trial (NCT00689221) failed to show any improvement. Thus, Cilengitide is not further developed as an anti-cancer [112].

THPs are also used for peptide receptor radionuclide therapy (PRRT), which combines a tumor-homing peptide with a radionuclide or radioactive isotope as the therapeutic substance [167]. Cholecystokinin (CCK) receptors bind to gastrin, a 33 amino acid peptide hormone, and CCK2 receptor is abundant in tumors [168]. Human colorectal and pancreatic tumors have been treated using ^111^In-labeled CCK8 and minigastrin (LEEEEEAYGWMDF), a peptide specifically designed to target CCK-2 receptors, in mice [43].

PRRT is used in clinics. [^177^Lu]-DOTATATE (Lutathera) (Novartis, Basel, Switzerland) (Table 3 for peptides in clinics) was approved by FDA in 2018 for PRRT for gastroenteropancreatic neuroendocrine tumors (GEP-NET) after showing improved survival in NETTER-1 Phase III clinical trial (NCT01578239) [169,170]. DOTATATE [DOTA-(Tyr^3^)-octreotate] is an 8 aa cyclic disulfide peptide with a covalently bonded DOTA bifunctional chelator [2,2′,2″,2‴-(1,4,7,10-tetraazacyclododecane-1,4,7,10-tetral) tetraacetic acid, tetraxetan]. The peptide octreotate (octreotide acid) mimics natural somatostatin targeting the somatostatin receptors (SSTR).

Another example of recently approved PRRT is [^177^Lu] Lu-PSMA-617 (Pluvicto) (Novartis), which targets prostate-specific membrane antigen (PSMA), thus showing efficacy in inhibiting prostate cancer growth [171,172].

**Table 3:** Peptides in clinics for cancer treatment and diagnosis.

Name	Company	Year	Targets	Used for	Refs.
Leuprorelin, Lupron, Viadur, Eligard, Fensolvi	Abbott Laboratories, Abbott Park, IL, USA	1985	GnRH receptor	Prostate cancer, breast cancer	[173,174]
Goserelin, Zoladex	TerSera therapeutics, Deerfield, IL, USA	1997	GnRH receptor	Prostate cancer, breast cancer, endometriosis	[175,176]
Octreotide, Sandostatin	Novartis, Basel, Switzerland	1998	Reduction of growth hormone	treat diarrhea or diarrhea associated with some types of cancer	[177]
Cetrorelix, Cetrotide	Merck Serono, Darmstadt, Germany	2000	GnRH receptor	In vitro fertilization	[175]
Abarelix, Plenaxis	Praecis Pharmaceuticals, Waltham, MA, USA	2003	GnRH receptor	Advanced prostate cancer	[9]
Degarelix, Firmagon	Ferring pharmaceuticals, Saint-Prex, Switzerland	2008	GnRH receptor	Advanced prostate cancer	[173,175,178]
Carfilzomib, Kyprolis	Onyx Pharmaceuticals, Newbury Park, CA, USA, and Amgen, Thousand Oaks, CA, USA	2012	Proteasome inhibitor	multiple myeloma	[179]
Netspot, ^68^Ga DOTATATE	Novartis, Basel, Switzerland	2016	SSTR	PET diagnostics of neuroendocrine tumor	[180,181]
Lutathera, ^177^Lu-DOTATATE	Novartis, Basel, Switzerland	2018	SSTR	PRRT for gastroenteropancreatic neuroendocrine tumors	[169,170]
Edotreotide gallium, ^68^Ga-DOTATOC	ITM Radiopharma, München, Germany	2019	SSTR	PET for neuroendocrine tumors	[182]
Detectnet, ^64^Cu-DOTATATE	RadioMedix, Houston, TX, USA	2020	SSTR	PET for neuroendocrine tumors	[183,184]
Gallium gozetotide, ^68^Ga-PSMA-11	Novartis, Basel, Switzerland	2020	PSMA	PET for recurrent prostate cancer	[185,186,187]
Pylarify, piflufolastat F18,	Lantheus, Billerica, MA, USA	2021	PSMA	PET for recurrent prostate cancer	[188,189]
Pluvicto, ^177^Lu-PSMA-617	Novartis, Basel, Switzerland	2022	PSMA	PRRT for metastatic castration-resistant prostate cancer	[171,172]

SSTR: somatostatin receptors, PSMA: the prostate-specific membrane antigen.

## 3. Peptide Design Strategies

In recent decades, there has been a notable increase in the availability of peptide drugs in the market. However, peptide drugs face challenges in formulation and delivery compared to small molecules, limiting their development. Factors such as shorter circulation half-lives, lower cell permeability, enzymatic degradation, and limitations in oral delivery hinder the efficient administration and absorption of therapeutic peptides [190,191]. Indeed, therapeutic peptides offer advantages such as high target specificity and low toxicity, making them promising candidates for drug development [192]. Overcoming the current limitations associated with their formulation and delivery would unlock their full potential, leading to the development of safer and more effective drugs. By improving the delivery mechanisms, enhancing stability, prolonging circulation half-lives, and optimizing routes of administration, the limitations of therapeutic peptides can be addressed, resulting in the development of advanced cancer therapies with improved clinical outcomes. Various design strategies have been developed to improve the effectiveness of bioactive peptides [193].

### 3.1. Cell-Penetrating Peptides

Cell-penetrating peptides (CPPs) are short peptides containing fewer than 30 amino acid residues, with a high content of basic amino acids like arginine and lysine. These peptides possess the remarkable ability to transport various cargo across cellular membranes while maintaining their functional integrity. CPPs can be utilized as either ACPs or THPs [194].

The first CPP, human immunodeficiency virus (HIV) TAT, can deliver the biologically active fusion protein to all tissue in mice [44]. Penetratin is a 16 aa CPP derived from *Drosophila* Antennapedia homeodomain and widely used in preclinical settings [45,46]. Transportan is a CPP derived from galanin, a natural peptide distributed throughout the nervous system [47]. 

The aforementioned p28 is also a CPP, which preferentially penetrates into cancer cells [16]. CPP p28 enhances the cytotoxic activity of temozolomide in the glioblastoma multiforme model [195]. Additionally, p28 can cross the blood–brain barrier (BBB) to enhance the efficacy of DNA-damaging agents by activating the p53-p21 axis [196].

### 3.2. Peptide Cyclization

Peptide cyclization involves the transformation of linear peptides into cyclic peptides, which helps mitigate proteolytic degradation caused by amino- and carboxypeptidases. This process masks both the N-terminal amino group and the C-terminal carboxyl group, resulting in enhanced stability against enzymatic degradation. Cyclic peptides also have a limited number of conformations in solution, allowing them to bind more efficiently to the active site of the desired target [197]. For example, cyclic peptides with the sequence cNGXGXXc, specifically targeting integrin α3β1, were found to enhance cell adhesion by selectively binding to the over-expressed integrin α3β1 in non-small lung cancer cells [50].

Cyclic peptides have been used for tough targets such as the Ras family GTPases [198]. NKTP-3, a cyclic D-peptide, inhibited the growth of A427 cells carrying the KRAS^G12D^ mutation by specifically targeting NRP1 and KRAS^G12D^. It also exhibited potent antitumor activity in xenograft models derived from A427 cells and primary lung cancer models driven by KRAS^G12D^, all while displaying no apparent toxicity [51]. KRpep-2d (Takeda, Tokyo, Japan) was developed from screening of cyclic peptides on phage displaying interaction to recombinant K-Ras^G12D^ [52]. LUNA18 (Chugai Pharmaceutical, Tokyo, Japan) is also a cyclic peptide targeting KRAS [105]. LUNA18 is currently tested in a Phase I clinical trial for solid tumors (NCT05012618) and is likely for progressing into Phase II [199].

### 3.3. Manipulation of the Amino Acid Sequence

Replacing partly or fully the L-amino acids with D-amino acids in peptide structures is a viable approach to enhance stability and decrease immunogenicity [200]. Examples include octreotide, a modified version of somatostatin with all L-amino acids replaced by D-amino acids, resulting in increased enzymatic stability and a longer plasma half-life [201]. Octreotide (Sandostatin) (Novartis) was first approved by FDA to reduce growth hormone in patients with acromegaly in 1998. Octreotide is used in clinics to treat the symptoms associated with metastatic carcinoid tumors (flushing and diarrhea) and vasoactive intestinal peptide (VIP) secreting adenomas (watery diarrhea). Octreotide acid (octreotate) is used as [^177^Lu]-DOTATATE (Lutathera) (Novartis) for PRRT in clinics. Octreotide conjugated with DOTA, DOTATOC (DOTA-[Tyr3]-octreotide), is extensively tested in clinical trials for diagnostic imaging as described in Section 4.2.

The use of D-amino acids in antifouling peptide biosensors and antimicrobial peptides has shown enhanced stability and activity. However, complete substitution with D-amino acids may lead to reduction in vivo activity and potential toxicity [202,203].

### 3.4. Peptides Conjugated with Polymers

The bioavailability and stability of peptide drugs can be improved through polymer–peptide conjugations. This approach involves attaching therapeutic peptides to polymers such as polyethylene glycol (PEG), poly(amidoamine) (PAMAM), poly(β-amino ester) (PAE), and natural polysaccharides, which leads to nanoscale self-assemblies and larger sizes that prevent renal filtration [204,205,206]. For example, A 40 kD PEG linked to the HVGGSSV peptide efficiently targets Tax-Interacting Protein 1 (TIP1), which is known to be overexpressed in human-invasive breast cancer cells [53,54].

### 3.5. Peptide-Assembled Nanoparticles

Peptides can be designed to self-assemble or be combined with polymeric molecules to create nanoparticles through non-covalent bonds. These nanoparticles have demonstrated attractive properties, including improved recognition of targeted cells, responsiveness to microenvironments, facilitation of internalization, and enhanced therapeutic effects [207]. X4-2-6, a PEG-modified 24-amino acid peptide analog of the second transmembrane helix of CXCR4, forms nanoparticles that inhibit CXCR4 function, prevent bone metastasis, and serve as a drug delivery system [55].

## 4. Peptides in Applications

### 4.1. Routes of Administration

**Figure 2:**
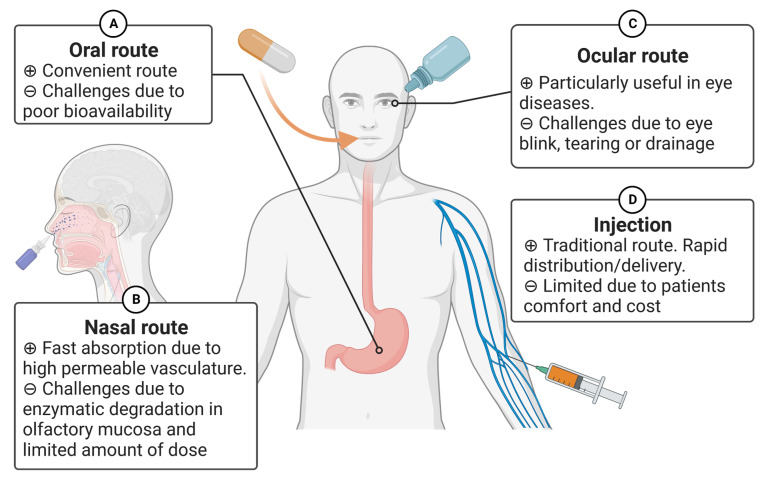
Routes of administration in humans. Comparison of four different routes of drug administration: (**A**) oral, (**B**) nasal, (**C**) ocular and (**D**) intravenous injection.

Recent advancements in biopharmaceutical engineering have resulted in the development of various drugs based on peptides or proteins [7,167,194,208,209]. The route of drug administration significantly affects its effectiveness as a treatment [210,211]. Currently, several peptide-based drugs are on the market. For instance, Leuprorelin (Abbott Laboratories, Abbott Park, IL, USA), Goserelin (TerSera therapeutics, Deerfield, IL, USA), and Cetrorelix (Merck Serono, Darmstadt, Germany) have been used for breast cancer and prostate cancer patients. They are designed as hormone analogs and rapidly absorbed following subcutaneous injection [173,174,175,176]. Although the traditional route of administering protein and peptide-based drugs using a needle and syringe is commonly practiced, it has certain limitations, such as patient comfort, cost, sterility, etc. In this section, we review alternative routes that have been proposed for peptides or proteins (Figure 2).

#### 4.1.1. Oral Route

This is one of the most convenient routes for administration of common drugs (Figure 2A). However, the administration of peptide drugs through this route poses challenges due to poor membrane permeability, denaturation of peptides due to an acidic environment in the stomach, and the susceptibility of peptides to enzymatic degradation in the gastrointestinal tract (GIT) [212,213]. In general, as the molecular weight increases, the permeability decreases. To overcome these limitations, several formulation technologies have been proposed. For example, to enhance the stability of peptide- or protein-based drugs, co-administered enzyme inhibitor(s) have been studied. Codelivery of therapeutic peptides with enzyme/protease inhibitors can inhibit degradation in the intestinal lumen, leading to greater absorption and bioavailability. Some examples of such inhibitors are soybean trypsin inhibitors, aprotinin, and leupeptin [214]. Aprotinin is currently withdrawn from the market due to the negative mortality trend [215]. To improve therapeutic peptide stability without undesirable adverse effects, it is crucial to select appropriate protease inhibitors based on the sequence/structure of therapeutic peptide drugs and to optimize a balance between the efficacy and safety of peptidase inhibitors.

The other type of co-administrative materials for peptide oral delivery have also been studied. Chitosan nanoparticles are proposed to use as a carrier for orally delivering peptides and vaccines. They possess remarkable biocompatibility, controlled release of peptides, and promote absorption in the GIT. Also, enclosed peptides within these nanoparticles can endure enzymatic degradation and withstand different pH conditions [216].

Various types of compounds with diverse chemical properties, including surfactants, fatty acids, medium chain glycerides, steroidal detergents, acylcarnitine and alkanoylcholines, *N*-acetylated α-amino acids and *N*-acetylated non-α-amino acids, and mucoadhesive polymers have been studied to enhance the intestinal absorption of large polypeptide drugs. Although some of these have been tested in clinical trials [217], the challenge of low bioavailability in peptide drugs persists, and a comprehensive breakthrough with wide-ranging applicability to various peptides has not been fully achieved yet.

#### 4.1.2. Nasal Route

The epithelium of the nose is loosely packed with high permeable vasculature. This route can deliver peptides by transport mechanisms, including passive diffusion, carrier-mediated transport, and transcytosis [208,218,219] (Figure 2B). In general, peptides administered via the nasal route demonstrate increased permeation and faster absorption when peptides have a lower molecular weight. Clinically, IM862 (Cytran Inc., Kirkland WA, USA), a synthetic dipeptide (L-glutamine L-tryptophan), was previously tested. IM862 is a naturally occurring peptide with antiangiogenic properties by reducing the production of VEGF [220,221]. In the animal models, the antitumor activity of IM862 was similar, irrespective of the route of administration, including intranasal, subcutaneous, intravenous, and intramuscular [117]. Due to its convenient administration and relatively high bioavailability (71%) after intranasal administration, the nasal route was chosen for human trials Phase III (NCT00002445) [117].

Despite the potential of this delivery route, there are several challenges associated with the nasal route of administration for peptides or proteins [219]. After administration, bioavailability is significantly affected by the peptides’ surface, size, lipophilicity, and pI (isoelectric point). The enzymatic degradation in the mammalian olfactory mucosa is also an important factor [219]. The limitation of this route is associated with the restricted amount of dosage that can be administered. Consequently, it still requires the development and formulation of a drug with a high dose capacity [222]. In spite of such limitations, several advantages of this pain-free and non-invasive administration are evident. Hence, delivering the peptides through the nasal route is a potential alternative to drug delivery strategies. 

#### 4.1.3. Ocular Route

This route is particularly useful in treating ocular malignant tumors such as intraocular melanoma (uveal) (Figure 2C) [192]. Drug administration through the eye presents challenges due to its natural processes, such as blinking, tearing, and drainage.

As mentioned above, the bioavailability of peptides or proteins is generally low in most of the non-invasive routes (e.g., oral and nasal) [223]. The other current limitation in delivering peptide- and protein-based drugs through noninvasive routes may arise from the considerable expense, which could restrict the number of economically feasible options. Although the dosage form of the non-invasive routes can be self-administered by patients, the manufacturing cost of peptide and protein drugs would be less compared to the traditional injections at healthcare facilities. While considerable progress has been made previously, further advancements in formulation technology, such as the development of new penetration enhancers, enzyme inhibitors, etc., are still needed.

### 4.2. Diagnostics—Imaging

Cancer detection and management have been facilitated by the utilization of various imaging modalities. Molecular imaging with targeted probes or contrast agents has proven to be a valuable tool in diagnosing various types of cancer. Advances in molecular imaging technology may increase the precision with which therapies can be implemented [224]. However, the efficacy of molecular imaging is dependent on the imaging modalities and probes/contrast agents employed to specifically target, detect, and visualize cancer biomarkers. Typical imaging modalities in oncology include magnetic resonance imaging (MRI), positron emission tomography (PET), computed tomography (CT), optical fluorescence imaging, and ultrasound sonography. Imaging probes or contrast agents are predominantly composed of target-specific molecules that are designed to recognize and bind to tumors, enabling their visibility in various imaging modalities [225]. They can also participate in metabolic pathways. In many cases, they are labeled with metals, radioactive, or fluorescent materials. Although hardware such as highly sensitive detection sensors or scanners are also important factors in molecular imaging, in this review, we focus on imaging probes, particularly peptide-based agents on MRI, and nuclear medicine techniques such as single-photon emission computed tomography (SPECT) and PET, and optical/near-infrared (NIR) imaging.

#### 4.2.1. MRI

MRI utilizes a high magnetic field and creates images by applying specific radiofrequency pulses, resulting in distinct signal patterns across various tissues. These patterns depend on the composition of the tissues, specifically the types and concentrations of nuclei within them. MRI is a powerful non-invasive imaging technique that takes advantage of a very high spatial and temporal resolution and can provide detailed molecular/cellular information when combined with a contrast agent. MRI contrast agents are molecules or particles that influence the relaxation of water protons. They can be T_1_- or T_2_-weighted, affecting the longitudinal or transverse relaxation times, respectively. T_1_-weighted contrast agents are mostly small gadolinium (Gd^3+^) or manganese (Mn^2+^) paramagnetic complexes, while the majority of the T_2_-weighted ones are iron oxide-based superparamagnetic nanoparticles [226,227]. Due to the lack of sensitivity of MRI, relatively high concentrations of a contrast agent (mM range) need to be injected to generate local variation in the signal intensity. To achieve a specific binding to the target biomarker, different approaches (e.g., nanoparticles, antibodies, or peptides) have been studied [228,229,230,231]. In general, the use of peptides instead of small molecules to target the same biomarker leads to stronger binding affinity and selectivity [232,233]. In addition, compared to proteins, peptides have advantages such as low immunogenicity, more resistance to enzymatic degradation, favorable pharmacokinetics, and biodistribution [232,234].

Gadolinium (III) is often used as an MRI contrast agent since it has seven unpaired electrons and a high magnetic moment [235]. Presently, to avoid free Gd (III) and nephrotoxicity, the approved Gd contrast agents are chelated with a total of eight nitrogen and oxygen from the chelator function as Gd^3+^-binding ligands [236]. Two of the most common Gd-based contrast agents are Gd-DTPA (diethylene triamine pentaacetic acid, Magnevist) and Gd-DOTA (Dotarem) [237]. Although they are useful and have been used clinically, their elimination half-life is relatively short (~1.3 h in humans) [238]. In addition to delivery strategies, by attaching targeting moieties, optimizing the half-life and pharmacokinetic properties of these contrast agents can substantially improve the imaging quality and eliminate repeated dose injections. The modifications with several antibodies [e.g., targeting VEGF, epidermal growth factor receptor (EGFR), or human epidermal growth factor receptor 2 (HER2)] have been proposed to improve the MRI contrast agents [239]. The Gd modifications with peptides for MRI applications have also been studied. For instance, peptides conjugated with DTPA (Gd) or DOTA (Gd) by a PEG linker improved the activity suggesting their potential application as diagnostic agents for MRI [56,240,241]. Tumor-homing peptide, such as CREKA [40], is promising for detecting cancer by MRI in vivo [240,241].

#### 4.2.2. SPECT

Radionuclides are unstable nuclides that emit α, β+/− charged particles, Auger electrons, and/or γ rays with radioactive decay processes. Radionuclides used in medical imaging can be categorized into two types based on their emissions; the first type consists of radionuclides that emit γ rays, which can be detected using SPECT. The second type includes radionuclides that primarily emit β+ rays, which can be detected through techniques such as PET [242]. Although the sensitivity, resolution, and fast acquisition of SPECT due to the solid-state detector technology provide a great impact on nuclear diagnosis [243], various carrier molecules to target tumors have been proposed. As proteins, radiolabeled trastuzumab and pertuzumab antibodies have shown high accumulation in tumor tissues [244]. In this case, the optimum timeframe for evaluating antibody-based imaging with favorable tumor-to-organ ratios is typically 3–5 days following administration. Given that the smaller molecular size can alter the clearance pathway and peptides have relatively shorter circulation time, peptides can be suitable molecules for imaging procedures [245]. Peptide-based probes for SPECT have been developed in several preclinical and clinical studies [246,247]. For example, RGD tri-amino acids peptide can specifically bind to the integrin αVβ3 receptors, which is highly expressed in several types of tumors. ^99m^Tc-PEG4-E[PEG4-c(RGDfK)]2 (^99m^Tc-3PRGD2) is proposed as an RGD containing SPECT radiotracer. Multicenter studies suggest that imaging with ^99m^Tc-3PRGD2 is sensitive for the detection of lung malignancies [248].

#### 4.2.3. PET

As noted above, the basic principle of PET is that proton-rich radionuclides decay by emitting positrons (β+), which subsequently travel a short distance and annihilate with an electron (β−) to create two 511-kilo electron volt photons that arise almost exactly 180 degrees apart [249]. The properties of some radionuclides that decay via β+ decay are shown in Table 4 [250,251].

Similar to SPECT, peptides have been used as carrier molecules to deliver these radionuclides for PET. Various labeling methods have been designed [209]. In clinical studies, various peptides have been explored for tumor diagnosis [232,252]. For example, peptides targeting somatostatin receptors (SSTR), the prostate-specific membrane antigen (PSMA), integrins, chemokines, urokinase-type plasminogen activator receptors (uPAR), and cholecystokinin receptors (CCK2-R) serve as notable examples [253,254,255,256,257,258]. Among them, Netspot (^68^Ga-DOTATATE) (Novartis) [180,181], Edotreotide gallium (^68^Ga-DOTATOC) (ITM Radiopharma, München, Germany) [182,259], and Detectnet (^64^Cu-DOTATATE) (Radio-Medix, Houston, TX, USA) [183,184] target SSTR and approved by FDA for PET in 2016, 2019, and 2020, respectively. Gallium gozetotide (Locametz, Illuccix, ^68^Ga-PSMA-11) (Novartis) [185,186,187] and Pylarify (piflufolastat F18, ^18^F-DCFPyL) (Lantheus, Billerica, MA, USA) [188,189,260,261,262] targets PSMA and is approved by FDA in 2020 and 2022, respectively.

Radionuclides-labeled peptide probes are important tools in molecular imaging using SPECT and PET. Given the extensive exploration of novel labeling strategies and the ongoing optimization of associated peptides, the advancement of peptide-based tracers will continue to be a pivotal area of focus in imaging research with radionuclides.

#### 4.2.4. Optical/NIR Imaging

Optical imaging incorporates multiple modalities, including bioluminescence imaging (BLI), fluorescence, and chemiluminescence [263]. They are often used in preclinical studies. Among them, fluorescence imaging has been used in clinical studies. In particular, fluorescent agents that are emitted in the NIR region (700–900 nm) enable sufficient depth of light penetration allowing for real-time surgical guidance. Imaging in the NIR region offers several advantages, such as the presence of minimal tissue absorbance, scattering, and autofluorescence. These unique features establish a minimal background, providing an ideal basis for incorporating tissue-specific contrast. Due to these properties, such as high sensitivity, contrast, and resolution, intraoperative fluorescence imaging is particularly well-studied for surgical applications. Accurate detection of visually hidden tumor lesions and tumor margins during surgery can lead to a significant impact on overall cancer survival/outcomes. Among various types of solid tumors, the highest positive margin rate following tumor resection in the US is oral cavity tumors [264]. Ovarian and prostate cancers had the highest positive margin rate prevalence in women and men, respectively. Bladder, thyroid, colorectal, kidney, lung cancer, and breast cancer are common types of tumors with the highest positive margin rates [264,265]. The substantial increase in the number of early-phase clinical trials for image-guided surgery with NIR agents reflects a significant potential to develop novel methods for visualizing and accurately identifying tumor margins in specific cancer types. Indocyanine green (ICG) is one of the most commonly used NIR dyes. While it has an excellent safety profile in humans, ICG is a non-specific agent that is rapidly cleared by the liver and excreted in bile [266,267]. Currently, most strategies for NIR dyes, including ICG, use conjugation technologies with targeting motifs such as peptides [268].

Because the positive margin rate following breast-conserving surgery (lumpectomy) is unignorably high, we developed a new near-infrared fluorescence imaging probe ICG-p28 by utilizing ICG labeled with the cell-penetrating peptide p28 carrying a tumor-targeting motif [16]. As described earlier in the sections of ACP and CPP, p28 crosses the BBB and selectively enters cancer cells in mouse models [195,196]. Due to such unique tumor-targeting properties, ICG-p28 was developed and tested in clinically relevant preclinical settings [269,270]. These studies demonstrated that intraoperative imaging with ICG-p28 accurately identified the tumor margins, improving tumor recurrence rate in multiple breast cancer animal models independent of the receptor expression status [269,270]. These results emphasize the importance of the imaging approach and its translational potential.

### 4.3. Targeting Delivery

Drug delivery is critically important for optimizing the efficacy of drugs while reducing toxic adverse effects. Several approaches have been proposed to deliver active compounds to the target sites [194,208]. In this section, we focus on peptide-based targeting delivery.

In passive targeting, peptides can accumulate at tumor sites by intrinsic characteristics of peptides such as size and charge and due to distinctive properties of the targeted sites such as local vasculature and lymphatic drainage. At tumor sites, nearby vasculature is generally leaky, and lymphatic drainage is impaired or absent [271]. Under such conditions, the enhanced permeation and retention (EPR) effect allows preferential accumulation of peptides within the tumor tissue. The concept of the EPR effect was first proposed in 1986 [272], and the EPR effect with a universal mechanism is a unique feature of solid tumors [273,274,275,276]. In hypoxic conditions or inflammation, blood vessels become more permeable. New blood vessels (neovascularization) in hypoxic tumors are generally leaky as they have large openings (200–2000 nm) [277,278].

In contrast, receptor-mediated approaches are active targeting strategies [279,280,281]. The abundant expression of peptide-binding receptors in human tumors highlights their potential as promising targets for selective anti-cancer drug delivery. Synthetic analog peptides of natural ligands are of significant interest as receptor-targeting entities due to their ability to exhibit high affinity, rapid internalization rates, and low immunogenicity. Several target candidates for this group of peptides, such as integrin receptors, HER2, and EGFR [280,281].

In addition, CPPs are also very efficient in delivering various molecules into cells [194]. Although the entry mechanism of CPPs into cells is still a matter of some debate, direct translocation and/or endocytotic cellular entry are often described as the entry mechanism of CPPs [282,283].

As described in the previous section, CPPs have significant potential to target delivery vehicles, and currently, ~2000 CPPs have been identified based on the CPPsite 2.0 database. An important challenge in cancer therapy involves the limitations of drug delivery to the target lesions by barriers such as the tumor microenvironment or the BBB. CPPs opened a new avenue to overcome such limitations.

### 4.4. Crossing the BBB with Peptides

**Figure 3:**
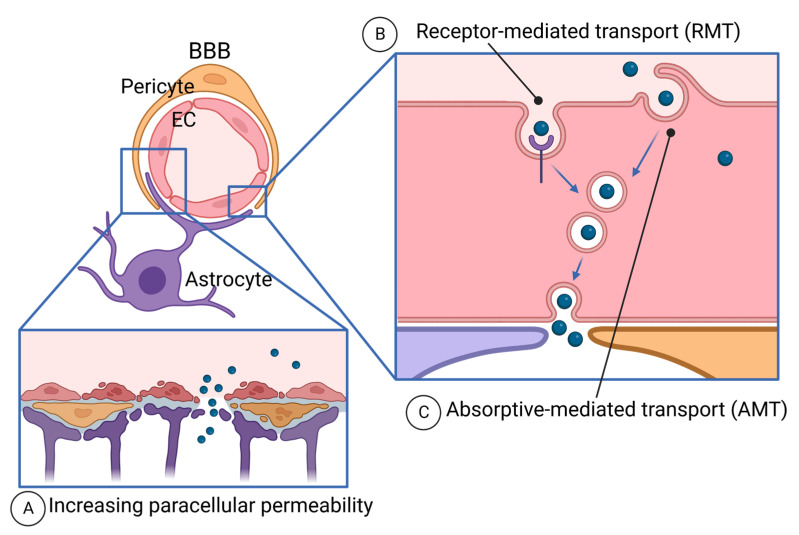
Routes of drug delivery crossing the BBB. (**A**) The BBB is formed by a tightly connected EC layer covered by pericytes and astrocyte end foot. To deliver the drugs via paracellular routes, junctions are disrupted by various methods described in Section 4.4.1. (**B**) While keeping the barrier intact, drugs can be delivered by receptor-mediated transport by conjugating drugs with ligand-mimicking peptides, as described in Section 4.4.2. (**C**) Drugs can be delivered by absorptive-mediated transport. CPPs and BBB shuttles use this pathway as described in Section 4.4.3.

The BBB is a unique microvasculature of the CNS, protecting the brain from harmful agents in circulation [284] (Figure 3). Due to the tightest protection by the BBB, drug delivery to the brain tumor is one of the big challenges of cancer therapies. Having tumor-targeting or vascular-targeting abilities of peptides, potential drug delivery to brain cancer (glioma and CNS lymphoma), and brain-metastasized cancer have been tested.

Drug delivery beyond the BBB is mediated by paracellular diffusion and transcellular route [285]. Paracellular diffusion is largely eliminated by tight junctions (TJs) and disruption of the BBB or temporal regulation of the BBB is necessary, as discussed below. The transcellular route uses the movement of molecules through the cells, passing through apical and basolateral membranes [286].

#### 4.4.1. Paracellular Diffusion and the BBB Disruption

To facilitate drug delivery to the brain, BBB disruption (BBBD) has been tested since the 1970s in animal models and clinical trials [218,287] (Figure 3A). Bradykinin is an endogenous peptide that opens the BBB for small molecules, such as sodium fluorescein, but not for bigger molecules, such as albumin [288]. Bradykinin and its analog RMP-7 selectively induce permeability in tumor vasculature in the brain, not in the normal brain, as its B2 receptor is abundant in tumor vasculature [289,290]. RMP-7 (lobradimil and cereport) (Alkermes, Dublin, Ireland) has been tested as a combination with carboplatin in clinical trials [113,114,115,116]; however, it failed to show improvement of the efficacy of carboplatin in a randomized controlled Phase II trial [115]. RMP-7 is not currently being investigated for BBBD purposes [287].

Non-peptide molecules such as hypertonic mannitol [291,292,293,294], alkylglycerols [295], regadenoson [296,297], and other device-based strategies such as cranial implantable ultrasound [298,299], hyperthermia [300,301], low-level laser treatment (LLLT) [302], magnetic resonance-guided laser ablation (MRgLA) [303], MR-guided focused ultrasound (MRgFUS) [304,305] has been tested, but progress has not been satisfactory [287].

More recently, targeted approaches of reversible regulation of the barrier and more selectively to the BBB have been taken. The BBB is characterized by a tightly packed monolayer of non-fenestrated endothelial cells (ECs) connected by TJs, adherens junctions (AJs), and gap junctions [284]. Brain ECs have a higher expression of TJ proteins compared to arterial and venous ECs [306]. Especially, Claudin-5 has gained attention as a gatekeeper of the BBB [307]. A peptidomimetic derived from Claudin-5 induces transient BBB permeabilization and facilitates brain uptake of MRI contrast agents in mice [57].

Cadherins are important adherens junction proteins. E-cadherin-derived peptides (e.g., HAV6) induce endothelial permeability and inhibit resealing of tight junctions [60]. These E-cadherin-derived peptides facilitate drug delivery to the brain in animal models [58] and inhibit the growth of medulloblastoma in mice [59]. But these peptides also open junctions in intestinal epithelial cells in vitro [308].

#### 4.4.2. Transcellular Route—Receptor-Mediated Transport (RMT)

The transcellular route of drug delivery is endocytosis-mediated transcytosis, which is absorptive-mediated transport (AMT) and receptor-mediated transport (RMT) (Figure 3B,C). AMT mediates the uptake of cationic molecules at the luminal surface of EC and the exocytosis of the molecules at the abluminal surface [309]. We will discuss this below regarding CPP. RMT is mediated by the interaction of ligands to the receptors on the luminal surface of the BBB, thereby, ligand-derived peptides have been extensively developed [310]. For this purpose, the receptors need to be abundantly expressed at the luminal surface of ECs, and selectively to the BBB [311,312,313]. Such receptors include the low-density lipoprotein receptor (LDLRs) [314], transferrin receptor (TfR1) [315], insulin receptor [316], and leptin receptor [317,318].

Among them, LDLR has been most studied for RMT [319,320]. LDLR plays an important role in lipoprotein transport across the BBB for the delivery of essential lipids to the brain [314]. Their natural protein ligands are apolipoproteins (Apo) A [321], ApoB [322], and ApoE [323]. The peptides derived from ApoB and ApoE have been tested for drug delivery across the BBB [61,322,323,324,325].

LDLR-related proteins (LRPs) mediate transcytosis of lactoferrin [326], melanotransferrin (p97) [64,327], receptor-associated protein (RAP) [328], tissue plasminogen activator (tPA) [329], and β-amyloid precursor (APP) [330]. Aprotinin is known to interact with LRPs [331] and 19 amino acid peptide Angiopep-2 (AngioChem Inc., Montreal, QC, Canada) was designed from the LRP-binding domain of aprotinin [332,333]. Angiopep-2 was conjugated with FDA-approved chemotherapeutic agents, such as doxorubicin (ANG1007) [334], etoposide (ANG1009) [334], paclitaxel (ANG1005) [335], and bioactive peptides [336]. Its conjugate with paclitaxel (ANG1005 or GRN1005) [337] showed good tolerance in Phase I clinical trials [118,119] and tested for the treatment with recurrent high-grade glioma in combination with bevacizumab (NCT01480583). Dual-targeting of liposomes modified with Angiopep-2 and tumor-targeting peptide derived from neuropilin (tLyP-1) showed successful drug delivery of VEGF siRNA and docetaxel for glioma-bearing mice [338]. 

Other peptides binding to LDLRs are the ApoE-derived peptide K16ApoE [61], AEP [62], RAP-derived peptide RAP12 [63], and melanotransferrin (MTf)-derived peptide [64]. Screening of peptides using phage display has been applied to find the peptides binding to receptors. Peptide-22 (VH434), found by phage display, interacts with LDLR and facilitates drug delivery to the brain in animal models [49,65]. Other peptides found by phage display for binding to LDLR or LRP1 are L57 [66], M1 [67], and LRPep2 [68].

Phage display has been widely used to define TfR-binding peptides. TfR-T12 and T7 were identified by phage display [69] and tested for drug delivery to the brains of rodents [339,340] and zebrafish [341]. The other peptides found in the phage display are B6 [70], CRT peptide [71], and NanoLigand Carriers (NLC) [72]. TfRB1G3 was designed from natural peptides called Cystine-dense peptides (CDPs), a mini-protein class with high affinity and low immunogenicity [342]. 

Leptin receptor is highly expressed on the BBB [317,318]. In the same year, Leptin30 (aa 82–111 of human leptin P41159) [74] and peptide Lep70–89 (aa 91–110 of P41159) were defined as a brain-targeting peptide from leptin. Lep70–89-modified liposomes exhibit cellular uptake via macropinocytosis in mouse brain endothelial cells [343]. Leptin30 showed gene delivery to the brain in mice [73]. Another peptide, g21 (aa 33–53 of mouse leptin P41160), also facilitates the delivery of nanoparticles modified with g21 to the brain in rats [75]. 

RGD peptide, the most widely studied adhesive peptide found as an integrin-binding motif from extracellular matrix proteins [157], also provides targeted delivery to tumor vasculature as the αvβ3 integrin is highly expressed in angiogenic endothelial cells in tumors [344,345,346,347]. Cyclic RGD (cRGD) or stapled RGD (sRGD) have been used to modify micelles or liposomes and have shown accumulation in orthotopic glioma in mice [348,349]. Liposomes modified with cRGD could deliver anti-cancer drugs such as paclitaxel, oxaliplatin, and doxorubicin across the BBB, inhibited glioma growth, and improved the survival of glioma-bearing mice [348,349,350]. Such tumor-targeting and brain-penetrating property of RGD peptide has been tested as imaging agents in clinical trials [351]. ^18^F Fluciclatide (AH111585) (GE Healthcare, Chicago, IL, USA) was tested for PET imaging following i.v. injection to detect solid tumors, including high-grade glioma, in the Phase II trial (NCT00565721). ^18^F-RGD-K5 (Siemens Molecular Imaging Inc., Knoxville, TN, USA) is also an RGD-based radiotracer for PET imaging. Biodistribution and safety of i.v.-injected ^18^F-RGD-K5 was tested in monkeys and humans in Early Phase I study (NCT00743353) [120]. Other RGD-based radiotracers tested in Early Phase I for PET/CT imaging are ^68^Ga-BNOTA-PRGD2 (NCT01806675), ^18^F-FPPRGD2 (NCT01806675), and ^68^Ga-RM26-RGD (NCT05549024).

For therapeutic purposes, DNX-2401 (Delta-24-RGD, tasadenoturev) (DNAtrix, Inc., Houston, TX, USA) has been tested for glioma patients in clinical trials. DNX-2401 is a tumor-selective, replication-competent oncolytic adenovirus. A Phase I, dose-escalation, biologic-end-point clinical trial was conducted by intratumoral injection (NCT00805376) [127]. In another Phase I trial (NCT01582516), DNX-2401 was administered by convection-enhanced delivery (CED), which is a local drug delivery to bypass the BBB. Successful tumor targeting and safety in the brain were confirmed [125]. In the Phase I trial (NCT03178032), infusion of DNX-2401 through a catheter placed in the cerebellar peduncle followed by radiotherapy showed promising reduction/stabilization of tumor size of pediatric patients with diffuse intrinsic pontine glioma (DIPG) [128]. DNX-2401 was further tested in the Phase I trial by intra-arterial infusion (NCT 03896568) [129], and the Phase I/II study as a combination of intratumoral delivery of DNX-2401 followed by i.v. anti-PD-1 antibody pembrolizumab in recurrent glioblastoma (NCT02798406) [126]. Although RGD potentially crosses the BBB, the BBB penetration is not the focus of these studies. 

VEGFR2 and Neuropilin-1 (NRP1) are important co-receptors for VEGF to mediate angiogenesis. A peptide screened for binding to the VEGF receptors, A7R [77] interacts with NRP1 but not with NRP2 or VEGFR2 [352,353]. Glycosylated A7R derivative is stable in serum, able to cross the BBB to deliver paclitaxel to glioma in mice, and improves survival of glioma-bearing mice [76].

Interleukin (IL)-13 receptor (IL-13R) α2 is highly expressed in glioma, thus is considered as a target for drug delivery. A peptide derived from IL-13 (IL-13p) is used to modify docetaxel-loaded nanoparticles and showed suppression of the growth of s.c. glioma in mice [78]. Phage display found another peptide, Pep-1, which binds to IL-13Rα2 [79]. Following i.v. injection, Pep-1 homes to both s.c. and orthotopic GBM xenografts in mice [79] and facilitates delivery of chemotherapy agent, cilengitide (CGL) loaded in Pep-1 conjugated liposome, to suppress the growth of s.c. glioma [80].

A peptide G7 was derived from opioid peptide MMP-2200 [81,82]. G7-modified nanoparticles (NPs) cross the BBB by several endocytotic vesicles and macropinocytotic processes [81]. 

Neurotropic viruses, snake neurotoxins, and bee venoms have received attention as they interfere with brain cells. Rabies virus glycoprotein (RVG) interacts with nicotinic acetylcholine receptor (nAChR) on neuronal cells to enable viral entry into neuronal cells [354]. 29 mer RVG-derived peptide [355] was conjugated with 9R to enable siRNA binding (RVG-9R), which enabled the delivery of siRNA to the brain [83]. An independently identified 43 mer RVG-derived peptide (RDP) [84] and 39 mer RDP [85] also successfully delivered the fused protein to the brain in mice. KC2S is a synthetic peptide derived from snake neurotoxins that bind to nAChRs [86]. Paclitaxel-encapsulated KC2S-micelles afforded robust inhibition of intracranial glioblastoma in mice [86]. CDX is derived from snake neurotoxin candoxin, which also binds to nAChR [87]. CDX-micelle could deliver paclitaxel and improve the survival of glioblastoma-bearing mice [87]. To improve the stability, replacing with D amino acid (resulting in DCDX) could deliver doxorubicin for glioblastoma-bearing mice [88]. Apamin is a peptide found in bee venom, crossing the BBB [356,357], and MiniAp-4 is a shorter peptide derived from apamin [89].

Several peptide shuttles have been found through in vivo phage display biopanning without aiming for a particular receptor. The most prominent example is that of TGN [90]. This sequence is actively transported across brain endothelial cells, and its brain selectivity suggests that the mechanism is receptor mediated. The brain delivery capacity of this shuttle is supported by enhanced therapeutic effects in glioblastoma and Alzheimer’s mouse models [358,359].

#### 4.4.3. Transcellular Route—Absorptive-Mediated Transport (AMT) and Brain-Penetrant Peptides

The mechanisms of drug delivery to the brain using cell-permeable peptides (CPPs) are not fully understood. It is believed that cationic CPPs bind to the luminal surface of the EC membrane and are endocytosed via AMT and exocytosed at the abluminal surface [309] (Figure 3C). Some CPPs have the ability to penetrate the BBB and are used for drug delivery [360,361].

TAT can deliver the biologically active fusion protein to all tissues in mice, including the brain, beyond the BBB [362,363]. After that, more selective CPPs were found and tested for drug delivery.

Penetratin is a 16 aa CPP, derived from the *Drosophila* Antennapedia homeodomain [45,46]. Doxorubicin conjugated with penetratin [91] or encapsulating receptor-targeted liposomes modified with transferrin and penetratin [361] cross the BBB after i.v. injection in rodents.

Transportan is a CPP derived from galanin, a natural peptide distributed throughout the nervous system [47]. M13 is a derivative of transportan, showing the delivery of M13-conjugated cisplatin into the brain in GBM-bearing mice [48].

Protegrins are small peptides with antimicrobial activity found in porcine leukocytes [364]. SynB1 and SynB3 are peptides derived from Progrin-1 (PG1) and can deliver conjugated doxorubicin to the brain via absorptive-mediated transport [365,366].

Glutathione crosses the BBB via carrier-mediated transport [367]. Glutathione PEGylated (GSH-PEG) liposomes (G-Technology) [368,369]and glutathione-coated nanoparticles [370] have been used for drug delivery across the BBB. 2B3-101 (2-BBB, Leiden, Netherlands) is a glutathione PEGylated liposomal methylprednisolone, an anti-inflammation drug, showing efficacy in reducing the severity of encephalomyelitis in mice [130]. 2B3-101 is further tested in an Open-label, Phase I/IIa, dose-escalating study in patients with solid tumors, brain metastases, or recurrent malignant glioma (NCT01386580).

A peptide CAQK, found by in vivo phage display screening in mice with acute brain injury, interacts with chondroitin sulfate proteoglycans, upregulated in the injured brain [92]. CAQK-coated nanoparticle successfully delivered siRNA to the injured site in the brain after i.v. injection [92]. Peptides interacting with lipids on the membrane can also mediate drug delivery to the brain. Gangliosides are glycosphingolipids, abundant in neuronal cells. G23 peptide, found by phage display targeting gangliosides [93,94], could promote the transport of nanoparticles across the BBB, and provide a targeting effect [371].

A peptide named PepH3, derived from Dengue virus type 2 capsid protein (DEN2C), crosses the BBB by receptor-independent AMT [95]. Other peptides promising for drug delivery across the BBB are N-methyl phenylalanine-rich peptide [96], phenyl proline tetrapeptide [97], non-canonical anionic peptide NegPep [98], Porphyrin [99], and a neurofilament-derived peptide NFL-TBS.40–63 [100,101].

Finally, bypassing the BBB by intratumoral injection, implantation of drug-releasing polymers, convection-enhanced drug delivery [372], and intranasal delivery [222,373]. Low-molecular-weight protamine (LMWP) is a cell-penetrating peptide used as a conjugate with nanoparticles to facilitate drug delivery to the brain via intranasal administration in rats [102].

## 5. Future Perspectives

In this review, we described peptide-based approaches for cancer treatment. Many of these peptides were identified from natural sources such as proteins from bacteria, plants, or animals/humans since nature generates functional materials in living systems in the form of proteins and peptides. Although they are quite useful, artificial intelligence (AI) and machine learning-based strategies without human bias have the potential to revolutionize and accelerate the discovery and design of peptide-based drugs. In recent years, AI and deep machine learning-based strategies have shown immense potential in the medical field (e.g., drug discovery) [374,375]. These models have the capability to generate data that extends beyond what we have in the training samples, enabling an effective and fast tool for exploring the extensive search space of high-dimensional data, such as peptide/protein sequences. Although we are currently in the process of enhancing our understanding of utilizing AI safely and its widespread adoption in clinical settings currently remains limited, a new generation of peptide-based agents may soon be among the most important elements in clinical management as the utilization of AI is rapidly developing and appeared to help to minimize human errors.

## 6. Conclusions

In this review, we reviewed original research articles, recent review articles, and information from ClinicalTrial.gov to summarize the uses of peptide-based agents as anti-cancer therapeutics, drug delivery as peptide-drug conjugates or modifying liposomes/nanoparticles, and diagnostics imaging. New strategies to develop and design peptides are still being tested in preclinical models for future development of peptide-based diagnostics or therapies for cancer patients [7,9,194,376,377].

## Figures and Tables

**Table 2:** Examples of peptides tested in clinical trials.

Name	Company	Type	Clinical Trials	Refs.
p28 (NSC745104)	CDG Therapeutics, Elk Grove Village, IL, USA	ACS	Phase I (NCT00914914, NCT01975116)	[28,29]
ALRN-6924	Aileron Therapeutics, Watertown, MA, USA	ACS	Phase I/II (NCT02264613), Phase I (NCT02909972, NCT03654716, NCT03725436, NCT04022876, NCT05622058)	[103,104]
LUNA18	Chugai Pharmaceutical, Tokyo, Japan	ACS	Phase I (NCT05012618)	[105]
E75 (Nelipepimut-S, HER2/Neu, NeuVax)	SELLAS Life Sciences, New York, NY, USA	Peptide vaccine	Phase I (NCT00841399, NCT00091286, NCT01532960), Phase I/II (NCT00791037), Phase IIb (NCT01570036), Phase III (NCT01479244)	[106,107,108,109,110].
iRGD, CEND-1	Cend Therapeutics, San Diego, CA, USA		Phase I (NCT03517176), Phase I/II (NCT05052567, NCT05121038), Phase 2 (NCT05042128)	[111]
Cilengitide™ (cRGDfV, EMD 121974)	ICENI Pharma, Edinburgh, UK		Phase III (NCT00689221)	[112]
RMP-7, Cereport	Alkermes, Dublin, Ireland		Phase I (NCT00001502, NCT00005602), Phase II (NCT00019422)	[113,114,115,116]
IM862	Cytran, Kirkland, WA, USA	anti-angiogenesis	Phase III (NCT00002445)	[117]
Angiopep-2	Angiochem Inc., Montreal, QC, Canada	Drug delivery	Phase I (NCT01480583)	[118,119]
^18^F Fluciclatide (AH111585)	GE Healthcare, Chicago, IL, USA	PET	Phase II (NCT00565721)	
^18^F-RGD-K5	Siemens Molecular Imaging Inc., Knoxville, TN, USA	PET	Early Phase I (NCT00743353)	[120]
^68^Ga-BNOTA-PRGD2		PET	Phase I (NCT01542073, NCT01527058)	[121]
^18^F-FPPRGD2		PET	Early Phase I (NCT01806675),	[122,123]
^68^Ga-RM26-RGD		PET	Early Phase I (NCT05549024)	[124]
DNX-2401 (Delta-24-RGD, tasadenoturev)	DNAtrix Inc., Houston, TX, USA	Drug delivery	Phase I (NCT00805376, NCT01582516, NCT03178032, NCT 03896568), Phase I/II (NCT02798406)	[125,126,127,128,129]
2B3-101	2-BBB, Leiden, The Netherlands	therapy	Open-label, Phase I/IIa (NCT01386580)	[130]

Examples of peptides tested in clinical trials are shown.

**Table 4:** Properties of commonly used PET radionuclides.

Nuclide	Half-Life (min)	Emission Type	Mode of Decay (%β)	Energy (MeV)
^11^C	20.3	β+	99.77	0.97
^13^N	10	β+	100	1.20
^15^O	2	β+	100	1.74
^18^F	110	β+	96.7	0.64
^64^Cu	762	β+/electron capture	17.87	0.66
^68^Ga	68.1	β+/electron capture	87.7	1.90
^124^I	60,192	β+/electron capture	11.0	2.14

## Data Availability

Not applicable.
